# Cardiac magnetic resonance imaging in patients with left bundle branch block: Patterns of dyssynchrony and implications for late gadolinium enhancement imaging

**DOI:** 10.3389/fcvm.2022.977414

**Published:** 2022-10-20

**Authors:** Antonia Petersen, Sebastian Niko Nagel, Bernd Hamm, Thomas Elgeti, Lars-Arne Schaafs

**Affiliations:** Department of Radiology, Corporate Member of Freie Universität Berlin and Humboldt-Universität zu Berlin, Charité – Universitätsmedizin Berlin, Berlin, Germany

**Keywords:** cardiac MRI, left bundle branch block (LBBB), strain analysis, artefacts, late gadolinium enhancement (LGE)

## Abstract

**Background:**

Left bundle branch block (LBBB) is a ventricular conduction delay with high prevalence. Aim of our study is to identify possible recurring patterns of artefacts in late gadolinium enhancement (LGE) imaging in patients with LBBB who undergo cardiac magnetic resonance imaging (MRI) and to define parameters of mechanical dyssynchrony associated with artefacts in LGE images.

**Materials and methods:**

Fifty-five patients with LBBB and 62 controls were retrospectively included. Inversion time (TI) scout and LGE images were reviewed for artefacts. Dyssynchrony was identified using cardiac MRI by determining left ventricular systolic dyssynchrony indices (global, septal segments, and free wall segments) derived from strain analysis and features of mechanical dyssynchrony (apical rocking and septal flash).

**Results:**

Thirty-seven patients (67%) with LBBB exhibited inhomogeneous myocardial nulling in TI scout images. Among them 25 (68%) patients also showed recurring artefact patterns in the septum or free wall on LGE images and artefacts also persisted in 18 (72%) of those cases when utilising phase sensitive inversion recovery. Only the systolic dyssynchrony index of septal segments allowed differentiation of patient subgroups (artefact/no artefact) and healthy controls (given as median, median ± interquartile range); LBBB with artefact: 10.44% (0.44–20.44%); LBBB without artefact: 6.82% (-2.18–15.83%); controls: 4.38% (1.38–7.38%); *p* < 0.05 with an area under the curve of 0.863 (81% sensitivity, 89% specificity). Septal flash and apical rocking were more frequent in the LBBB with artefact group than in the LBBB without artefact group (70 and 62% versus 33 and 17%; *p* < 0.05).

**Conclusion:**

Patients with LBBB show recurring artefact patterns in LGE imaging. Use of strain analysis and evaluation of mechanical dyssynchrony may predict the occurrence of such artefacts already during the examination and counteract misinterpretation.

## Introduction

A left bundle branch block (LBBB) occurs when conduction through the left bundle branches is slowed down or completely absent, resulting in delayed depolarization of the left ventricular (LV) myocardium (1). There are many underlying causes, and LBBB is rather common with a prevalence that increases with age (2, 3). Patients with LBBB have an increased risk of suffering from a major cardiovascular event, and even in asymptomatic patients LBBB is likely to be associated with adverse myocardial remodelling (4, 5). Patients with LBBB may benefit from cardiac resynchronization therapy (CRT); however, the response to CRT depends in part on the degree of mechanical dyssynchrony and the extent of any underlying structural heart disease (6). For this reason, cardiac magnetic resonance imaging (MRI) with late gadolinium enhancement (LGE) imaging is becoming increasingly important in treatment planning and risk stratification of patients with LBBB since it allows diagnostic assessment of cardiac function, mechanical dyssynchrony, the extent of scar tissue and characterization of ischemic and non-ischemic cardiomyopathy in a single examination (7–9). LGE might also be an additional risk stratifier for the choice of therapy and implantation of defibrillation devices (7, 10). Delayed excitation leads to characteristic abnormalities of the cardiac cycle in up to 80% of affected patients. Such abnormalities include septal flash (the rapid deflection of the septum toward the free left ventricular wall at the onset of systole) and apical rocking (rocking movement of the apex due to dyssynchronous contraction of the left ventricle) and can be detected by cinematographic (CINE) imaging (11, 12). Additional MRI strain analysis may be helpful to quantify regional wall motion abnormalities. Strain parameters, such as the systolic dyssynchrony index (SDI), have been proposed to describe the dyssynchronous excitation of the LV in LBBB with regard to changes in global LV function parameters such as ejection fraction and ventricular volumes (13–17). Thus, when planning treatment of patients with LBBB using cardiac MRI, it is of particular importance for accurately estimating dyssynchrony and the amount of scar tissue that LGE images are not degraded by artefacts. While it is well established that arrhythmia in general or a delay in conduction is a challenge for cardiac MRI due to the risk of trigger artefacts or inappropriate myocardial nulling during LGE imaging, little is known about the specific influence of an LBBB on image properties (18). Therefore, the aim of the present study is twofold: first, to identify and describe patterns of artefacts that might occur in cardiac MRI of patients with LBBB compared to a healthy control group. Second, to define strain parameters or mechanical features that are associated with the occurrence of such artefacts.

## Materials and methods

### Study population

In this internal review board (IRB)-approved study (application number: EA4/192/21), we retrospectively screened all cardiac MRI examinations performed by our centre from 2015 to 2021 for patients with a LBBB, left anterior hemiblock or left posterior hemiblock in an ECG obtained during the period of hospitalisation in which the cardiac MRI examination was performed. A waiver for informed consent was granted by IRB due to the retrospective design of the study. Patients were included in further analysis based on the 2021 European Society of Cardiology criteria, i.e., when the reference ECG showed a widened Q wave, R wave, S wave (QRS) complex of > 120 ms and any other ECG characteristics of an LBBB (3). At the time of image acquisition, all patients were in sinus rhythm. For the purpose of comparison, a control group of patients who were referred for cardiac MRI due to non-specific thoracic symptoms was formed, in which both the cardiac MRI and the further cardiac diagnostic work-up showed no evidence of any pathological cardiac or cardiovascular findings and in which the reference ECG showed no signs of a conduction delay. Common inclusion criteria for patients and healthy controls were: age of 18 or older and a complete cardiac MRI dataset including CINE and LGE imaging in long and short axes. Clinical information such as cardiovascular risk factors and pre-existing cardiovascular conditions were extracted from the patients’ records.

### Cardiac magnetic resonance imaging

Cardiac MRI was performed either on a 1.5T or a 3T MRI system (Magnetom Aera or Magnetom Skyra, Siemens Healthineers, Erlangen, Germany). Acquisition of localizers was followed by retrospectively gated 2D steady-state free precession (SSFP) pulse sequences in double-angulated long-axis (2, 3, 4-chamber) and contiguous short-axis slices from the level of the annulus of the mitral valve to the LV apex (typical parameters: TR 39 ms, TE 1.2 ms, flip angle 69°, FOV 360 mm × 292 mm, matrix 192 × 125). Slice thickness was 5 mm for long-axis acquisition and 8 with 2 mm interslice gap for short-axis acquisition. Reconstructed temporal resolution was 35–44 ms. Late gadolinium enhanced images in long-axis (2, 3, 4-chamber) and short-axis views covering the whole LV were acquired 10–12 min after administration of 0.15 mmol/kg gadobutrol (Gadovist, Bayer AG, Leverkusen, Germany) using an inversion-recovery-prepared T1-weighted gradient echo sequence with a manually adjusted inversion time (TI) based on the TI scout as well as a parallel utilised phase-sensitive inversion-recovery (PSIR)-based reconstruction algorithm (typical parameters: TR 920 ms, TE 3.3 ms, TI 270 ms, flip angle 25°, FOV 360 mm × 292 mm, matrix 256 × 156 pixel spacing 1.45 mm × 1.45 mm, slice thickness 8, 2 mm interslice gap).

### Image analysis

Cardiac MRI datasets were evaluated by two board-certified radiologists with eight (*blinded*) and over fifteen (*blinded*) years of experience. For allocation of abnormalities and strain analysis, the LV was divided into segments based on the 17-segment model of the American Heart Association (19). CINE images of all included datasets were screened for signs of mechanical dyssynchrony, and the presence of a septal flash or apical rocking was noted. The TI scout and LGE images were evaluated by both readers in consensus with regard to inhomogeneous myocardial nulling and presence of LGE. The suppression of myocardium in the TI scout was defined as inhomogeneous if there was nulling but not of the entire myocardium at a single inversion time. An abnormality (i.e., inhomogeneous myocardial suppression) identified on LGE images was defined as an artefact only if it could not be reproduced in an intersecting second plane and if the distribution and extent were not consistent with typical characteristics of (non-)ischaemic LGE or if there were no matching features in other sequences (e.g., thinning or reduced kinetics of the myocardium in SSFP images) and also no interference from any other typical artefacts such as breathing or motion artefacts (20). True myocardial LGE was assessed in terms of its distribution and affected segments while both readers were blinded to the initial report.

### Strain analysis

Semi-automatic analysis of circumferential strain was performed using cvi42^®^ [Release 5.13.5 (2190), Circle Cardiovascular Imaging, Alberta, Canada]. The endocardial and epicardial contours were automatically registered by the software on short-axis CINE images covering the left ventricle. All contours were checked by two experienced readers. Whenever manual correction of the contours was necessary, it was performed by consensus by the readers. The global SDI (SDI_*global*_), defined as the standard deviation of the segmental time to maximum strain for segments 1–16 normalised to the length of the cardiac cycle and given in time percentage, was used to determine the severity of LV dyssynchrony and to correlate dyssynchrony with the frequency of artefacts identified in LGE imaging (21).

To further account for LV dyssynchrony due to delayed free wall contraction and associated septal deformation due to stretching in early systole, we investigated two additional new SDI:

(1) Septal SDI (SDI_*septal*_): defined as the standard deviation of the segmental time to maximum strain for segments 2, 3, 8, 9, and 14 normalised to the length of the cardiac cycle and given in time percentage.
(2) Free wall SDI (SDI_*free wall*_): defined as the standard deviation of the segmental time to maximum strain for segments 5, 6, 11, 12, and 16 normalised to the length of the cardiac cycle and given in time percentage.

The time required for strain analysis was less than 5 min and did not differ between patients and controls.

### Statistical analysis

For statistical analysis, three subgroups were defined: (1) patients with no inhomogeneous suppression on TI scout and no LGE imaging artefacts (LBBB_*no artefact*_), (2) patients with inhomogeneous suppression on TI scout and/or LGE imaging artefacts (LBBB_*artefact*_), and (3) healthy controls. Demographic data, QRS width, left ventricular function parameters and systolic dyssynchrony indices were tested for normal distribution within each subgroup (LBBB with and without inhomogeneous or incomplete suppression of the myocardium, controls) using the Shapiro–Wilk test, homogeneity of variances was asserted using Levene’s Test. In case of normal distribution of parameters, a one-way ANOVA was conducted, and Tukey’s or Games-Howell test was performed as a *post hoc* test depending on whether a variance equality could be assumed or not. If normal distribution could not be assumed for subgroups, non-parametric testing was performed with the Kruskal–Wallis test and Dunn’s test was performed as a *post hoc* test. Multiple comparisons were corrected for using the Bonferroni-Holm method. Systolic dyssynchrony indices in patients with LGE were compared using a *t*-test. If a significant difference was found between the subgroups, a receiver operating characteristic (ROC) curve analysis was performed to measure the diagnostic ability of the respective strain index. The optimal cut-off for separating LBBB_*artefact*_ and LBBB_*no artefact*_ was determined using the Youden index. A χ^2^-test for association was conducted between LBBB_*artefact*_ and LBBB_*no artefact*_ and occurence of apical rocking and septal flash. A *p*-value < 0.05 was considered statistically significant. Descriptive statistics are given as median (median ± interquartile range). SPSS 27 for Windows (IBM Corporation, Armonk, USA) was used for statistical analysis.

## Results

### Demographics and clinical patient characteristics

A total of 55 patients (18 female) with LBBB and 62 (27 female) controls were included. The median age of patients with LBBB was 57 years (34–80) whereas the median age of controls was 28 years (18–38). A summary of demographic and clinical data as well as global left ventricular function parameters, separately for patients and controls, can be found in Table 1. Although not statistically significant (*p* = 0.24), the median duration of the QRS complex was longer in LBBB_*artefact*_ (154 ms, 113–195 ms) than in LBBB_*no artefact*_ (139 ms, 108–171 ms), median duration of the QRS complex in controls was 88 ms (80–96 ms) Regarding global left ventricular function, LVEF was significantly lowered in LBBB_*artefact*_ when compared to LBBB_*no artefact*_ and controls (each *p* < 0.05). Concomitantly, LVEDV and LVESV were significantly higher in LBBB_*artefact*_ when compared to both other groups (each *p* < 0.05).

**TABLE 1:** Demographics, clinical characteristics, and summary of imaging-based parameters.

	LBBB_*artefact*_	LBBB_*no artefact*_	Controls
*n*	37	18	62
Age (years)	60 (43–76)^#^	54 (19–89)^#^	27 (15–39)
Male (*n*)	22 (59%)	15 (83%)	35 (56%)
Clinical characteristics			
Coronary heart disease	12^#^	8^#^	0
Non-ischemic cardiomyopathy	13*^#^	1	0
Hypertension	17^#^	7^#^	8
Smoking	9	1	11
Dyslipidaemia	7	6^#^	5
Obesity	7	1	6
Diabetes mellitus	7^#^	2^#^	0
QRS width (ms)	154 (113–195)^#^	139 (108–171)^#^	88 (80–96)
Left ventricular function parameters			
Heart rate (bpm)	71 (55–87)	66 (41–92)	72 (56–88)
LVEDV (ml)	235 (145–325)*^#^	180 (114–246)	133 (80–186)
LVESV (ml)	150 (46–254)*^#^	75 (27–123)	46 (21–71)
LVSV (ml)	90 (36–144)	107 (52–162)	89 (55–123)
LVEF (%)	37 (7–67)*^#^	59 (31–87)	67 (60–74)
LV myocardial mass (g/m^2^)	77 (41–113)^#^	66 (52–80)^#^	58 (47–69)
LGE all	15 (41%)	8 (44%)	0
LGE ischemic	10 (27%)	7 (39%)	0
LGE non-ischemic	5 (14%)	1 (6%)	0
Dyssynchrony analysis			
SDI_global_ (segments 1–16)	10.16% (2.16–18.16%)^#^	7.2% (1.2–13.2%)^#^	4.57% (2.57–6.57%)
SDI_septal_ (segments 2, 3, 8, 9, 14)	10.44% (0.44–20.44%)*^#^	6.82% (-2.18–15.82%)^#^	4.38% (1.38–7.38%)
SDI_free wall_ (segments 5, 6, 11, 12, 16)	3.65% (-0.35–7.65%)	4.67% (1.67–7.67%)	3.45% (1.45–5.45%)
Septal flash	23 (62%)*	6 (33%)	0
Apical rocking	26 (70%)*	3 (17%)	0

Overview of demographics, clinical characteristics and imaging-based parameters of patients (LBBB_*artefact*_/LBBB_*no artefact*_) and controls.

The upper part of the table shows demographics, clinical characteristics, left ventricular function parameters and LGE pattern (ischemic/non-ischemic) for both groups of LBBB and healthy controls. Results for analysis of mechanical dyssynchrony based on strain analysis and qualitative analysis of CINE images are shown in the lower part of the table.

Values are presented as median (median ± interquartile range), *p < 0.05 vs. LBBB_*no artefact*_, ^#^*p* < 0.05 vs. controls.

### Inversion time scout and late gadolinium enhancement imaging

**FIGURE 1:**
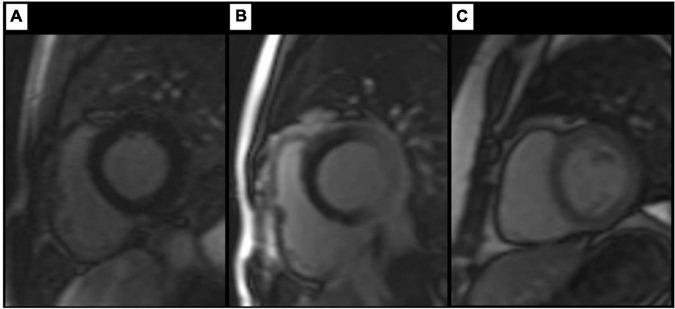
Findings on TI scout images. Artefact-free TI scout **(A)** in a healthy control. Images **(B,C)** show different degrees of inhomogeneous myocardial nulling in patients with LBBB, resulting in a pronounced, C-shaped nulling of the septal and anterior wall segments. Review of supplemental sequences revealed no morphologic correlate in any of the cases shown.

**FIGURE 2:**
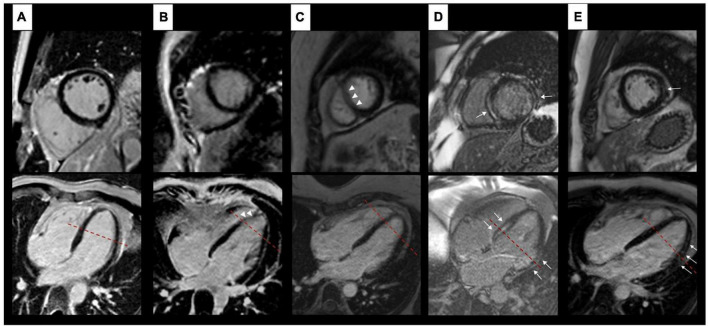
Findings on LGE images. Artefact-free images of a healthy control **(A)**. Columns **(B,C)** show examples of artefacts in patients with LBBB each resulting in an amorphous signal increase within the myocardium (white arrow heads) without a correlate in an intersecting plane. Examples of true LGE (white arrows) in patients with LBBB are shown in columns **(D,E)**. Here, both planes show an intramyocardial (septum) and subendocardial (free wall) signal increase **(D)** respectively a typical subepicardial signal increase **(E)**. The red dashed lines depict the respective intersection of both planes shown.

In 37 of 55 patients with LBBB (67%), there was no homogeneous nulling of the left ventricular myocardium in the TI scout (Figure 1). In these cases, a signal discrepancy between the septum and LV free wall was evident, resulting in a C-shaped suppression of the septum and anterior wall while large portions of the lateral and inferior wall were not nulled at this specific TI. The choice of a different TI in the scout also allowed nulling of the remaining myocardium with a signal difference consistently being visible between the aforementioned C-shape and the remaining segments. Artefacts on LGE images resulting in inhomogeneous myocardial suppression were identified in 25 of 55 patients (45%), all of whom also had inhomogeneous nulling in TI scout. Artefacts on LGE images were found either in the septal segments (17 of 25 patients, 68%) or in the segments of the LV free wall (8 of 25 patients, 32%). LGE artefacts resulted in areas with a patchy or amorphous signal increase within the myocardium that could not be assigned to a specific layer (subepicardial, intramyocardial, or subendocardial) or a coronary territory. Review of slices perpendicular to the affected image slice and of supplemental sequences identified no correlate in any of the 25 cases and therefore there were no cases where an artefact was present in both intersecting planes. Median inversion time was 273 ms (231–316 ms) for slices with evidence of artefacts. In 18 of those 25 (72%) cases artefacts persisted when utilising PSIR. Inhomogeneous myocardial nulling on the TI scout alone with no artefacts on consecutive LGE images was found in 12 of 55 patients (21%). Examples of LGE artefacts are shown in Figure 2. In the control group, no inhomogeneous myocardial nulling was found on the TI scout, and no comparable artefacts were identified on LGE images.

### Analysis of mechanical dyssynchrony

**FIGURE 3:**
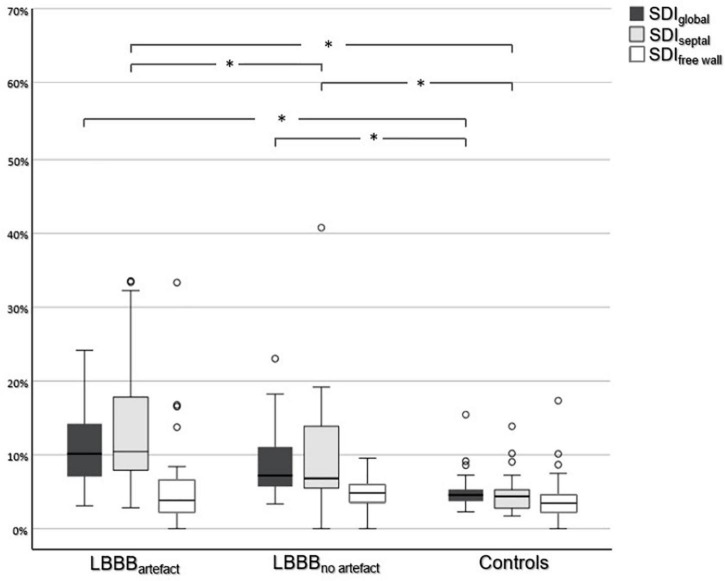
Comparison of systolic dyssynchrony indices. Comparison of SDI_*global*_, SDI_*septal*_, and SDI_*free wall*_ between all three subgroups. An asterisk (*) indicates a statistically significant difference (*p* < 0.05).

A summary of strain analysis and additional parameters of mechanical dyssynchrony is given in Table 1. Solely SDI_*septal*_ was able to differentiate between all three subgroups regarding the extent of left ventricular dyssynchrony (Figure 3). SDI_*septal*_ was highest in LBBB_*artefact*_ (10.44%, 0.44–20.44%), followed by LBBB_*no artefact*_ (6.82%, -2.18–15.82%) and controls (4.38%, 1.38–7.38%) (each *p* < 0.05). SDI_*global*_ was highest among LBBB_*artefact*_ with 10.16% (2.16–18.16%). SDI_*global*_ was generally able to differentiate between patients and controls (each *p* < 0.05), but could not distinguish between LBBB_*artefact*_ and LBBB_*no artefact*_ and therefore could not estimate the occurrence of artefacts in LGE imaging. SDI_*free wall*_ was highest among LBBB_*no artefact*_ with 4.67% (1.67–7.67%), but could not distinguish between the different groups. Regarding the occurrence of artefacts, ROC analysis revealed an area under the curve of 0.863 for SDI_*septal*_ with an optimal cut-off value of 7.36% (81% sensitivity, 89% specificity). Furthermore, SDI_*septal*_ was able to distinguish between LBBB_*artefact*_ and LBBB_*no artefact*_ in patients with LGE (*p* < 0,05), whereas SDI_*global*_ and SDI_*free wall*_ were not able to differentiate between these subgroups if LGE was present. All dyssynchrony indices did not significantly differ between patients with LBBB and LGE and patients with LBBB without LGE (*p* = 0.077–0.993). Septal flash and apical rocking occurred more often in LBBB_*artefact*_ with 70 and 62%, respectively, versus 33 and 17% for LBBB_*no artefact*_ (each *p* < 0.05). None of the controls showed these features of dyssynchrony. There was no significant difference between patients with LBBB and LGE and patients with LBBB without LGE in terms of occurrence of septal flash and apical rocking (*p* = 0.37 respectively *p* = 0.944).

## Discussion

The results of our study show that artefacts are common in LGE imaging of patients with LBBB and exhibit recurring patterns. While artefacts in TI scout and LGE images may be identified as such by trained observers, they are potential sources of error in clinical routine when imaging findings are reported by possibly less experienced readers, and this may have an impact on further treatment planning especially when the extent of scar tissue is then overestimated (22, 23).

Cardiac MRI is generally prone to artefacts due to motion, breathing and arrhythmia. In LGE imaging data acquisition is usually timed to diastole, with the heart exhibiting less motion, resulting in a homogenous nulling of the myocardium. There are two possible mechanisms that may lead to artefacts when patients with LBBB are imaged with an inversion pulse-prepared, segmented gradient echo (GRE) sequence (24, 25). First, inversion pulse preparation relies on the synchronisation of cardiac phases between the time of application of the inversion pulse and the time of read-out. However, because the septum and LV free wall are in slightly different phases of the cardiac cycle in patients with LBBB, it is likely that portions of the myocardium are not uniformly covered by the inversion pulse and thus are unevenly nulled during the read-out. Second, k-space sampling may be compromised in that the septum may be in different positions between the acquisition of different k-space segments despite sufficient ECG triggering. Established techniques to reduce artefacts in LGE imaging due to cardiac motion are the reduction of the FOV in the phase-encoding direction or, more commonly found, the use of single-shot inversion recovery balanced SSFP, single-shot spoiled GRE sequences and phase sensitive inversion recovery sequences (18, 26, 27). Further ways to reduce motion-induced artefacts have been proposed based on image acquisition during end-systole, and this may also be a possible approach in patients with LBBB given that in 72% of the cases in our collective artefact reduction using PSIR was unsuccessful (28, 29). Before utilising single-shot sequences with a generally lower spatial resolution or technically more complex systolic acquisition schemes, it seems desirable to determine the need for the use of such techniques early in the examination and before contrast medium is administered. Patients with LBBB show typical patterns of mechanical dyssynchrony and can therefore be identified early during the MR examination during the assessment of global function (11, 30, 31). A significantly higher proportion of patients in LBBB_*artefact*_ exhibited apical rocking and septal flash than in LBBB_*no artefact*_, suggesting that the risk for the occurrence of artefacts can be assessed already based on these two characteristics. However, a segmental and therefore more precise analysis of the extent of mechanical dyssynchrony can be achieved using strain analysis. Rutz et al. reported that a circumferential SDI (analogous to SDI_*global*_ in the present study) was highest among patients with LBBB and identified most severe dyssynchrony compared to controls and patients with myocardial infarction (13). While this agrees with our results, SDI_*global*_ did not differentiate between LBBB_*artefact*_ and LBBB_*no artefact*_. However, the newly investigated parameter SDI_*septal*_ showed a significant difference between these two subgroups and thus allowed differentiation. A possible explanation is that septal movement in patients with LBBB has both active and passive components, and delayed contraction of the LV free wall thus has a more marked effect on the apical segments of the septum than on its basal segments (32, 33). The data of the present study suggest that qualitative assessment of dyssynchrony (i.e., “septal flash” and “apical rocking”) already during CINE imaging can help to identify “patients at risk” while strain analysis with SDI_*septal*_ could further assist in identifying those patients prone to artefacts, the latter also with respect to the comparatively short computing time and a sufficiently good inter-study reproducibility (21). Consideration must be given to the fact that strain analysis by MRI is not widely used in institutions. Therefore, it can be assumed, based on the data of this work, that already the analysis of the TI scout and detection of septal dyskinesia on CINE images are an alternative guide in the evaluation of possible artefacts in LGE imaging.

To the best of our knowledge, this is the first study that has systematically analysed the occurrence of specific artefacts in this relatively common form of conduction delay. However, despite the potential utility of our results for routine clinical practice, some limitations need to be considered. First, due to the retrospective design of the study, it was not possible to form different subgroups (LBBB in ischemic or non-ischemic heart disease, LBBB of other aetiology) with sufficiently large numbers of patients for a more differentiated analysis of possible factors influencing dyssynchrony. Comparable studies included groups of 20–43 patients (1, 11, 14, 34). Secondly, and also due to the retrospective design, alternative MRI pulse sequence techniques such as single-shot inversion recovery balanced SSFP or single-shot spoiled GRE sequences could not be evaluated regarding their potential for artefact avoidance. Lastly, the patients in the control group, which was formed for the purpose of analysing occurrence of artefacts in uncompromised conduction, were not age- and sex-matched, hence any differences in global systolic function may also be due to age- or sex- differences alone.

## Conclusion

Patients with LBBB show recurring artefact patterns on cardiac MRI in both TI scout and LGE images, which may compromise the quality of cardiac MRI but also of subsequent reporting if readers are not familiar with these artefacts. The qualitative and quantitative analysis of left ventricular dyssynchrony may assist estimate the occurrence of such artefacts already during the MR examination and may help avoid misinterpretation.

## Data availability statement

The datasets used and/or analysed during this current study are available from the corresponding author on reasonable request.

## Ethics statement

This study was approved by the Internal Review Board of Charité-Universitätsmedizin Berlin (Germany, Berlin). Application number: EA4/192/21. A waiver for informed consent was granted by IRB due to the retrospective design of the study.

## Author contributions

AP made substantial contributions to the design of the study, the strain analysis, interpretation of data and drafting the manuscript. SN and BH made substantial contributions to drafting the manuscript. TE made substantial contributions to the design of the study, analysing late gadolinium enhancement images, the interpretation of data and drafting the manuscript. L-AS made substantial contributions to the design of the study, strain analysis, analysing late gadolinium enhancement images, the interpretation of data, drafting the manuscript and has agreed to ensure that questions related to the accuracy or integrity of any part of the work, even ones in which the author was not personally involved, are appropriately investigated, resolved, and the resolution documented in the literature. All authors contributed to the article, agree to be accountable for their contributions, and have approved the submitted version.

## Supplementary material

Supplementary material 1 Four-chamber steady-state free precession CINE images of a patient with LBBB. Typical features of dyssynchronous contraction in LBBB are evident: (1) rapid movement of the interventricular septum during early systole (“septal flash”) and (2) movement of the apex toward the interventricular septum during early systole and backward movement of the apex toward the free wall during the ejection phase (“apical rocking”).

## Abbreviations

GRE: gradient echo
LBBB: left bundle branch block
LGE: late gadolinium enhancement
LV: left ventricular/left ventricle
PSIR: phase sensitive inversion recovery
SDI: systolic dyssynchrony index
SSFP: steady-state free precession.
